# Editorial: Network neuroscience in neuropsychiatric disorders

**DOI:** 10.3389/fnhum.2026.1964327

**Published:** 2026-08-26

**Authors:** Dafa Shi, Kun Zhao, Yuhui Du, Hongfu Sun, Gen Yan

**Affiliations:** 1 Department of Radiology, The Second Affiliated Hospital of Xiamen Medical College, Xiamen, China; 2 Center for Artificial Intelligence in Medical Imaging, School of Artificial Intelligence, Beijing University of Posts and Telecommunications, Beijing, China; 3 School of Computer and Information Technology, Shanxi University, Taiyuan, China; 4 School of Engineering, University of Newcastle, Newcastle, NSW, Australia

**Keywords:** brain networks, functional connectivity, network neuroscience, neuroimaging, neuromodulation, neuropsychiatric disorders, structural connectomics

Neuropsychiatric and neurological disorders have long been conceptualized through a localizationist framework, in which specific symptoms are mapped onto discrete lesions or regions. Over the past two decades that view has been progressively complemented by a network-based understanding of brain function, in which cognition, behavior, and their disruption emerge from the coordinated activity of distributed, interacting neural systems (Bassett and Sporns, 2017). This Research Topic, “*Network neuroscience in neuropsychiatric disorders*,” was assembled against that background. Its seven contributions apply resting-state and task-based functional MRI, functional near-infrared spectroscopy (fNIRS), magnetic resonance spectroscopy (MRS), magnetoencephalography (MEG), and diffusion-based structural connectomics to a deliberately heterogeneous set of conditions—from somatic and neuromodulatory interventions in healthy volunteers to spinal cord injury, insomnia, Alzheimer's disease, diabetic retinopathy, and Parkinson's disease. Together, these studies illustrate both the explanatory power and the methodological fragility of network-level approaches to brain–behavior relationships, with the aim of making their shared strengths and limitations mutually visible.

A first cluster of articles examined how exogenous interventions reshape large-scale networks. Zheng et al. combined resting-state fMRI metrics (ALFF, ReHo) with JuSpace neurotransmitter atlas correlation to show that a single session of meridian-sinew therapy, a manual and scalp-acupuncture-based somatic intervention rooted in Traditional Chinese Medicine, enhanced prefrontal activity while suppressing putamen–insula regional homogeneity in healthy volunteers, changes that co-localized spatially with serotonergic, endocannabinoid, dopaminergic, and cholinergic receptor/transporter distributions. This multimodal strategy—linking macroscale functional change to microscale neurochemical architecture—exemplifies an increasingly influential paradigm for inferring candidate neurotransmitter mechanisms from non-invasive imaging. Joshi et al. used a combined fMRI–MEG design to demonstrate that anodal transcranial direct current stimulation over the left dorsolateral prefrontal cortex in early Alzheimer's disease reduced pathological hyperconnectivity between prefrontal seed regions and the posterior default-mode network, increased task-related activation in memory-relevant cortex, and enhanced theta–gamma phase-amplitude coupling in the entorhinal cortex—a convergence of resting, task, and oscillatory markers that strengthens confidence in tDCS as a cognition-enhancing intervention with a traceable network signature.

A second group of studies characterized disease-related network reorganization without any intervention. Zou et al. applied fNIRS to spinal cord injury and found that interhemispheric sensorimotor cortex connectivity, rather than prefrontal–motor connectivity, tracked residual sensorimotor function, proposing a lateralized-compensation account that reframes disconnection after spinal cord injury as potentially adaptive rather than purely pathological. Dai et al. used MEGA-PRESS spectroscopy to link reduced thalamic GABA+ to anxiety and fatigue severity in insomnia disorder, offering a neurochemical rather than purely connectivity-based window onto network dysregulation, while explicitly cautioning that macromolecule co-editing limits the specificity of the GABA+ signal. This neurochemical framing is not unique to insomnia: in temporal lobe epilepsy, MRS-derived GABA has similarly been proposed as a biomarker for lateralizing the epileptogenic side and tracking anti-seizure drug effects in MRI-negative patients (Wu et al., 2023). Song et al. extended seed-based connectivity analysis to diabetic retinopathy, showing that striatal subregions—particularly the ventral striatum, dorsal caudate, and rostral putamen—exhibited more extensive disconnection from salience, cognitive-control, visual, and sensorimotor networks in retinopathy than in diabetes without ocular complications, implicating a dual burden of microvascular damage and visual deprivation acting through a putative “visual–striatal loop.” Collectively, these three studies show that non-invasively detected network alterations can carry clinically meaningful information about impairment severity even without overt structural damage.

The one purely conceptual contribution, by Jerath and Malani, proposed the “default spatial representation” (DSR) framework, arguing that disorders as clinically disparate as contralateral neglect, anosognosia, disorders of consciousness, autism spectrum disorder, Alzheimer's disease, schizophrenia, and depersonalization/derealization disorder share a common underlying vulnerability: disintegration of an internally generated, default-mode-network-anchored representation of self-in-space. While speculative and not derived from new empirical data, this synthesis usefully reframes the Topic's more disease-specific findings within a transdiagnostic network architecture, and its proposed biomarkers—default mode, salience, and frontoparietal network coupling—resonate directly with the connectivity metrics used elsewhere in this Research Topic.

Finally, Sinzinger et al. provided a necessary methodological counterpoint. Applying a rigorously documented structural connectomics pipeline together with support-vector-machine and graph-neural-network classifiers to a large Parkinson's Progression Markers Initiative cohort, they found no connection-, node-, or whole-brain-level differences surviving correction for multiple comparisons, and near-chance classification regardless of tractogram-filtering strategy or connectivity metric. Rather than a result to dismiss, this is a cautionary benchmark: structural connectomic findings can be highly sensitive to pipeline configuration and cohort characteristics, and reported group differences may not generalize across protocols. Notably, this does not imply that combining brain networks with machine learning is inherently unrewarding. Rather, such a strategy adds value only when the underlying network signal is itself robust. A functionally derived analog illustrates this: an ALFF-based regional radiomics similarity network, submitted to a machine-learning classifier, distinguished patients with Parkinson's disease from controls with good accuracy in both a primary and an independent external validation cohort, with discriminative edges mapping onto default-mode and sensorimotor networks, among others (Shi et al., 2023). Set against Sinzinger et al., this contrast suggests that combining network analysis with machine learning can meaningfully enhance diagnostic value, but only once the underlying network is built from a sufficiently reproducible, disease-sensitive signal.

Taken as a whole, the seven contributions to this Research Topic illustrate two complementary facets of contemporary network neuroscience: on one hand, its capacity to generate mechanistically interpretable, multimodal, and clinically correlated findings across an unusually broad range of conditions; on the other, the discipline's ongoing struggle with reproducibility, modest sample sizes, and pipeline-dependent results. We put interventional studies, disease-characterization studies, a theoretical framework, and a methodological stress test side by side in the hope that readers will take network findings seriously as biomarkers while staying alert to the analytic choices that shape, and occasionally manufacture, the networks being reported. Larger and harmonized multi-site cohorts, pre-registered and openly shared pipelines, and the kind of multimodal triangulation across functional, structural, spectroscopic, and electrophysiological measures seen in several papers here would all help move the field forward.
